# Optimization of Polymer Processing: A Review (Part I—Extrusion)

**DOI:** 10.3390/ma15010384

**Published:** 2022-01-05

**Authors:** António Gaspar-Cunha, José A. Covas, Janusz Sikora

**Affiliations:** 1 Institute of Polymers and Composites, University of Minho, Campus de Azurém, 4804-533 Guimarães, Portugal; jcovas@dep.uminho.pt; 2 Department of Technology and Polymer Processing, Faculty of Mechanical Engineering, Lublin University of Technology, Nadbystrzycka 36, 20-618 Lublin, Poland; janusz.sikora@pollub.pl

**Keywords:** polymer processing, single screw, twin screw, injection moulding, blow moulding, thermoforming, optimization, artificial intelligence

## Abstract

Given the global economic and societal importance of the polymer industry, the continuous search for improvements in the various processing techniques is of practical primordial importance. This review evaluates the application of optimization methodologies to the main polymer processing operations. The most important characteristics related to the usage of optimization techniques, such as the nature of the objective function, the type of optimization algorithm, the modelling approach used to evaluate the solutions, and the parameters to optimize, are discussed. The aim is to identify the most important features of an optimization system for polymer processing problems and define the best procedure for each particular practical situation. For this purpose, the state of the art of the optimization methodologies usually employed is first presented, followed by an extensive review of the literature dealing with the major processing techniques, the discussion being completed by considering both the characteristics identified and the available optimization methodologies. This first part of the review focuses on extrusion, namely single and twin-screw extruders, extrusion dies, and calibrators. It is concluded that there is a set of methodologies that can be confidently applied in polymer processing with a very good performance and without the need of demanding computation requirements.

## 1. Introduction

Polymer processing is an important industrial activity that converts raw materials, such as polymers, polymer compounds, polymer blends, composites, and nanocomposites, into useful products mostly for applications in packaging, building and construction, mobility, electrical and electronics, medical, agriculture, household, leisure, and sports. For example, in 2019, more than 55,000 European companies (plastics raw materials producers, plastics converters, recyclers and machinery manufacturers in the EU28 Member States) employed over 1.5 million people, and converted 50.7 Mt of plastics [1]. A progressively more sustainable and better performing range of polymer systems, together with increasingly more efficient and intelligent extrusion, injection moulding, blow moulding, and thermoforming—the most important processing techniques for thermoplastics–are paramount to create or improve products with more advanced performances and functionalities.

Thermoplastics processing typically involves three functional steps: plasticization of a solid polymer (usually supplied in pellet form), flow and shaping of the melt, and cooling. Thus, an understanding of polymer processing requires a good knowledge of heat transfer, melt rheology, fluid mechanics, and morphology development, among others. In the case of reactive extrusion, plasticization is combined with chemical reactions (polymer synthesis and/or modification) into a single process. In their seminal book on polymer processing, Tadmor and Gogos [2] proposed a structural breakdown of polymer processing into elementary steps, based on the principles of transport phenomena, fluid mechanics, heat and mass transfer, polymer melt rheology, solid mechanics, physics and chemistry of polymers and mixing, which provide the basic tools for quantitatively analysing polymer processing.

It is well recognized that the geometry of the processing equipment, the operating conditions selected, and the properties of the polymer system being processed determine the resulting morphology of the part and hence its practical performance. This has fostered extensive experimental investigation with the aim of obtaining a good understanding of the physical, thermal, rheological, and chemical processes developing during polymer processing. Historically, the screw extraction experiments carried out by Maddock [3] were particularly relevant to shed light on plasticating extrusion. Once physical models of the underlying phenomena were available, they were translated into mathematical descriptions, either analytical or numerical, depending on the assumptions and simplifications made. Currently, modelling of polymer processing is well developed [2,4,5,6], with simulation software devoted to various processing techniques being commercially available. These programs solve the governing equations that describe the phenomena developing along the various individual process stages, coupled to the relevant boundary conditions (operating conditions and equipment geometry) and constitutive equations for the polymer properties. The resulting predictions provide a description of how the process will perform under the conditions defined, which is obviously very useful to both processors and equipment/tool manufacturers. In some cases, the morphology of the part (macromolecular/fibre orientation, crystallization rate, size of the spherulites, etc.) can be predicted from the knowledge of the thermomechanical process parameters, but the link to the end-use engineering properties entails multi-scale modelling [7], which is still very costly computationally.

The direct use of these simulations for practical process troubleshooting, setting the operating conditions, defining a screw profile, designing an extrusion die, an injection mould, or a plastic bottle, would require tackling the inverse problem, i.e., to solve the set of governing equations of the process in order to the geometrical and operational variables, while prescribing the required performance as boundary condition(s). This is complex and usually mathematically ill-posed, as there is no unique relationship between cause and effect. Thus, in practice, four alternative methodologies can be adopted:

1. Use the simulation tools on a trial-and-error basis. This is obviously expensive and inefficient and relies on the capability of the user to input progressively more appropriate boundary conditions.
2. Develop specific design approaches, i.e., using the modelling equations in a pre-arranged sequence. Examples include methods to design extruder screws [8] or extrusion dies [9].
3. Adopt an optimization procedure, whereby the process modelling package is used judiciously by an optimization algorithm, in order to define a “best” solution, or a Pareto optimal solution (see below). Practical polymer processing problems generally involve multiple, often conflicting, criteria (for example, maximizing output while minimizing viscous dissipation and mechanical energy consumption in plasticating single-screw extrusion); hence this approach is usually labelled as multi-objective optimization.
4. Perform data-driven optimization, which consists in the use of Artificial Intelligence (AI) techniques to explore the search space based on experimental or computational data [10,11].

Some of these methodologies were already presented in several reviews analysing the application of optimization techniques in polymer processing. Kasat et al. [12] discussed the application of Genetic Algorithms (GAs) in polymer science and engineering, stressing the existence of multiple objectives and constraints that must be dealt with simultaneously. Particularly in the area of chemical reactions during polymerization, obtaining a polymer with a desired molecular weight requires satisfying simultaneously various objectives, such as minimizing the reaction time as it implies lower cost and minimizing the concentration of side products, as they decrease the product properties. Oduguwa et al. [13] extended the idea of applying Evolutionary Algorithms (EA) to the manufacturing industry, which includes polymer processing. The main justification given for the use of this type of algorithms comes from the fact that the traditional methods frequently employed to solve complex real-world problems tend to miss a more efficient exploration of the search space, becoming trapped in sub-optimal regions, while simultaneously they are often computationally expensive. Methodologies based on the use of a population of points (solutions), such as EAs, can overcome this situation, as these points can evolve simultaneously towards the Pareto optimal front and thus access the global optimal solutions. Oduguwa et al. [13] also refer to the difficulties that optimization approaches face in being accepted in industry. Recently, Nastaj and Wilczyński [14] addressed the optimization and scale-up of single- and twin-screw extrusion. However, they focused on methodologies developed by specific authors, and the optimization concepts discussed were somewhat limited. Thus, in general, previous reviews on the optimization of polymer processing excluded several important technological or optimization aspects, are very limited in terms of the analysis performed, and even ignore several important published contributions.

After introducing some important concepts of multi-objective optimization, the present review covers the application of optimization methods to solve real problems in polymer processing, encompassing extrusion, injection moulding, blow moulding, and thermoforming. Due to the extension of the analysis, the work is divided into two parts. The present part 1 focuses on extrusion, namely single and twin-screw extruders, extrusion dies, and calibrators. The most important contributions to the field are identified and future trends are discussed.

Some relevant databases were used to explore the open literature, namely Science Direct, Google Scholar, Scientific.net, and publishers’ databases such as Springer, Wiley, MDPI, etc. The following topics/keywords were used in the search: optimization of polymer extrusion, optimization of single-screw extrusion, optimization of twin-screw extrusion, optimization of extrusion dies, optimization of extrusion calibrators, design of polymer extruders, and design of extrusion calibrators. The references cited in many papers were also an important source of information. The contributions found were either selected or excluded based on the effective use of consistent optimization methodologies applied to the polymer processes under consideration. Papers by the same authors that added nothing new were discarded. The present review is addressed to those process engineers, researchers, and experts in polymer processing who wish to gain knowledge in optimizing these technologies.

## 2. Need for Optimization in Polymer Processing

**Figure 1:**
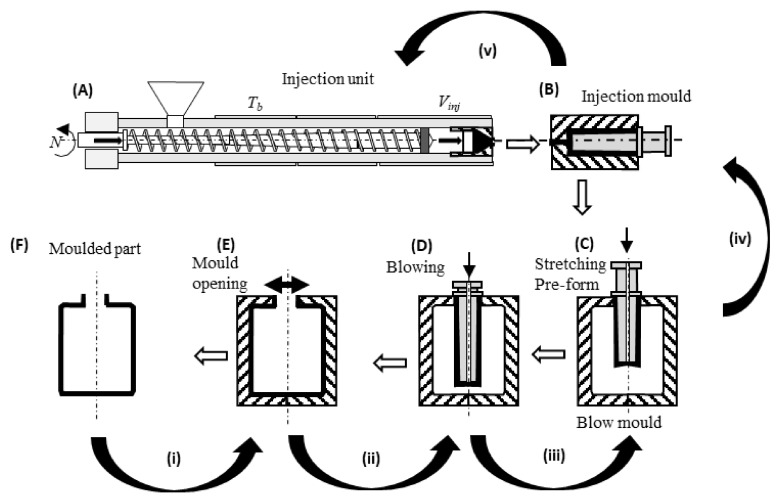
Optimization of Injection Stretch Blow-Moulding. (**A**–**F**) illustrate the process steps. Open arrows follow the process sequence; curved arrows follow the optimization sequence.

The practical need for optimization, namely in what concerns the process, the design variables, the modelling requirements, and the objectives to be attained, will be illustrated with an example dealing with Injection Stretch Blow-Moulding, a technique widely used for the manufacture of bottles for plain and carbonated drinks (and dealt with in greater detail in Part 2 of this review). Figure 1 illustrates the main production steps (Figure 1A–F, following the open arrows). An injection unit (A) fills the cavity of an injection mould (B) to produce a pre-form. This is then transferred to a blow-mould (C) where it is stretched and blown (D) against the contours of the mould cavity. Once the container is sufficiently cold, the mould opens (E), the part is removed and a new production cycle is initiated. Modelling this process in order to predict the performance of the part for a given set of input conditions (bottle design, operating conditions, equipment geometry, material properties) typically entails the numerical modelling of each of its individual steps, followed by coupling them through appropriate boundary conditions. Depending on the physical process models considered and consequent ability of the numerical routines, the thickness profile of the product, its morphology, and the mechanical performance could be predicted.

Such a sophisticated tool would be used quite inefficiently if conventional means were adopted. For example, if the aim is to produce a bottle with a minimum weight and a maximum thickness uniformity, the computer model will be used on a trial-and-error basis, with the operator progressively fine tuning the operating conditions and/or bottle design until attaining a satisfactory result. A much more efficient strategy would be to consider again the modelling sequence and solve the inverse problem, whereby the bottle characteristics are imposed as boundary conditions and the equations are solved in order of the operating conditions. This would imply approaching the process backwards, from step F to step A, as indicated by the curved arrows in Figure 1. However, this is mathematically ill-posed, as there are no unique relationships between cause and effect.

Alternatively, an optimization problem can be defined, consisting of two objectives (minimize bottle weight and maximize its thickness uniformity) and a restriction (the minimum thickness must be higher than a pre-defined value). The decision variable is the 3D thickness distribution of the part that results from all prior process steps, which constitutes a huge decision variable space. Either the optimization can proceed following steps (i) to (v) in Figure 1, or the system can be considered as a whole and all steps can be optimized simultaneously.

**Figure 2:**
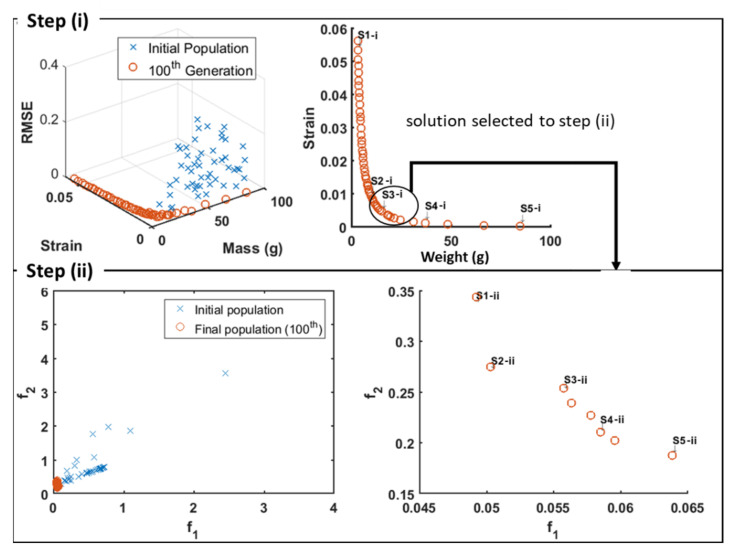
Typical results for a blow-moulding optimization: (i) optimization of bottle thickness profile; (ii) optimization of pre-form thickness profile before blowing. For example: solution S3-i is selected for step (ii), from which a new Pareto set is obtained.

Figure 2 shows results obtained for the optimization of steps (i) and (ii). In step (i), the aim is to define the thickness profile of the bottle that minimizes its weight, the maximum strain under a given load, and the thickness uniformity in terms of a parameter RMSE. Using EAs, an initial population of solutions is generated randomly, evolving until the 100th generation. From the analysis of the Pareto front, solution S3-i was selected. The following optimization problem concerns the blowing phase (here considered to take place after stretching the pre-form), i.e., the optimization of the thickness profile of the preform that produces the optimal bottle thickness profile found in the first step. For that purpose, the mean (*f*_1_) and the maximum (*f*_2_) errors between the optimal thickness distribution and the thickness distribution of the parison must be minimized. More details on this optimization can be found elsewhere [15,16].

## 3. Multi-Objective Optimization

The aim of optimization is to find the best set of decision variables, i.e., a solution that optimizes an objective function on a given search space, often in the presence of equality and/or inequality constraints, with the main purpose of approaching that solution to a global optimum [17]. Without loss of generality, in the case of a maximization problem, the mathematical formulation is the following:

(1) $$\begin{array}{l} \mathrm{maximise}\;\;f(x_{i})\;\;\;\;\;\;\;i=1,\ldots ,N\\ \mathrm{subject}\;\mathrm{to}\;\;g_{j}(x_{i})\geq 0\;\;\;j=1,\ldots ,J\\ \;\;\;\;\;\;\;h_{k}(x_{i})=0\;\;\;k=1,\ldots ,K \end{array}$$

where *f* is the objective function of the *N* parameters *x_i_*, *g_j_* are the *J* (*J* ≥ 0) inequality constraints, and *h_k_* are the *K* (*K* ≥ 0) equality constraints.

In the absence of a systematic procedure, a traditional way of finding the best possible solution consists of performing a statistical and/or regression analysis based on experimental or computational results. From a set of data, it is possible to deduce a mathematical model relating the objective function (*f*) with the decision variables (*x_i_*). From this model, an approximation to the optimal solution can be found both graphically and mathematically. This simple approach relates linearly the objectives with the decision variables. The quality of the solution depends strongly on the number of solutions available to construct the model. Consequently, more elaborated models can be deduced if more solutions are available. This type of regression method has different forms of being identified in the literature: design of experiments, response surface, statistical analysis, data fitting, etc.

**Figure 3:**
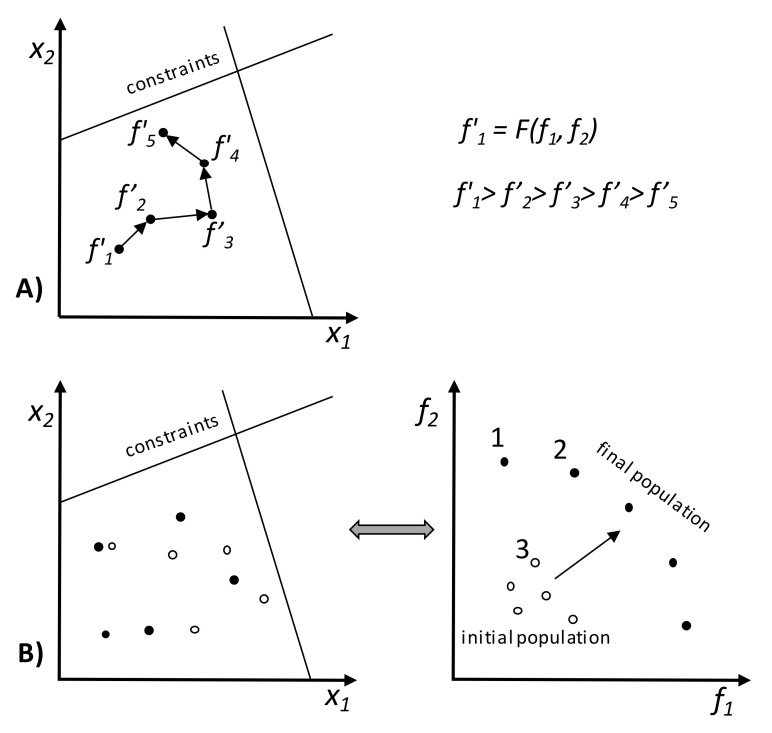
(**A**) Single-objective optimization versus (**B**) multi-objective optimization.

Another kind of methodology uses some type of information to perform the search. In most classical algorithms, the problem is solved starting with a solution generated randomly in the search space and, by means of a moving rule in a unidirectional direction based on the use of local information, the algorithm progresses point-by-point to find the best solution (Figure 3A). This new, optimized solution will be the starting point for the next step, where the same procedure is repeated a number of pre-defined times. If more than one objective exists, they must be aggregated into a single objective. The differences between the available algorithms rely on the way this search direction is defined. Two types of such methods exist: direct and gradient-based. In the first case, the search is only guided based on the values of the objective function and constraints, e.g., simplex search, pattern search, and conjugate direction methods. These methods are usually slow, requiring a high number of function evaluations. The second type involves the use of information concerning the first and/or second derivative of the objective function’s values and/or constraints, e.g., steepest descent and conjugate gradient methods. The use of derivatives hastens convergence, but these methods are unable to deal with non-differentiable and discontinuous problems. The calculation of the derivatives must be possible, which does not often happen in real problems [17]. These methods face other difficulties: (i) the convergence is strongly dependent on the initial solution chosen, (ii) the solution found is often stuck in a local sub-optimum, and (iii) the algorithm is unable to deal with a discrete problem and cannot take properly into account its multi-objective nature. In fact, real-world optimization problems (such as polymer processing) can comprise linear and/or non-linear objective functions and constraints, discrete and/or continuous variables, stochastic or deterministic inputs, and single or multiple objectives. Thus, the choice of the algorithm to use will depend strongly on the problem features [17].

Most (if not all) real optimization problems are multi-objective, i.e., it is necessary to satisfy simultaneously several performance measures (objectives), which are often conflicting. Additionally, their relative importance to the process may be subjective and can be dealt with in different ways. Mathematically, a multi-objective optimization problem can be defined as [18,19]:

(2) $$\begin{array}{l} \mathrm{maximise}\;\;f_{m}(x_{i})\;\;\;\;\;\;\;i=1,\ldots ,N;\;m=1,\ldots ,M\\ \mathrm{subject}\;\mathrm{to}\;\;g_{j}(x_{i})=0\;\;\;\;j=1,\ldots ,J\\ \;\;\;\;\;\;\;\;h_{k}(x_{i})\geq 0\;\;\;k=1,\ldots ,K \end{array}$$

where *M* is the number of objectives.

The various objectives can be taken into account a priori, a posteriori, or iteratively. In the first case, the optimization takes place after the decision maker (DM) defines the relative importance of the objectives using, for example, weights or goals. The performance of the solutions can be obtained through the use of aggregation functions, e.g., weighted sum, weighted product, or weighted Tchebycheff metric [20]. Then, a traditional single-objective methodology can be used to find the optimum, as illustrated in Figure 3A. A weighted sum is simple, but not only is it difficult to set the weight vectors to obtain a Pareto-optimal solution in a desired region of the objective space, it does not allow one to find certain Pareto-optimal solutions in a nonconvex and/or discontinuous objective space. The weighted Tchebycheff metric guarantees finding all Pareto-optimal solutions, assuming that the ideal solution in this multidimensional space (z*) is known. However, some weaknesses also exist: (i) the minimum and maximum values of the objectives and of z* must be known; (ii) for a small number of objectives, not all Pareto-optimal solutions are obtained; and (iii) as the number of objectives increases, the problem becomes non-differentiable [18,19]. The second alternative consists in optimizing simultaneously all the objectives without considering beforehand the preferences of the DM. The results will be a set of solutions denoted as Pareto set where two spaces of interest exist, instead of a single one as before, i.e., the decision variables and the objectives domain, as depicted in Figure 3B. Thus, the aim of multi-objective optimization is to find feasible solutions where all objective functions are optimized. These solutions are incomparable to each other, since it is not possible to state that one is better than another in all objectives simultaneously. The Pareto solutions are the set of non-dominated solutions (the full circles in Figure 3B). In the figure, Solution 2 is better than Solution 3 in both objectives; thus, Solution 3 is dominated by Solution 1. The same does not happen when comparing Solutions 1 and 2, as none of these solutions dominates the other. In this case, the selection of a solution can only be made using additional preference information that must be provided a posteriori by the DM [20]. Finally, the optimization and choice of solutions steps can be made iteratively and interleaved, i.e., the optimization algorithm provides alternative solutions to the decision maker, who indicates his/her preferences, and the optimization algorithm runs again taking into account this information. The process is repeated until a satisfactory solution (or solutions) is/are found. The decision making can be performed by humans and/or by computer algorithms [21].

**Figure 4:**
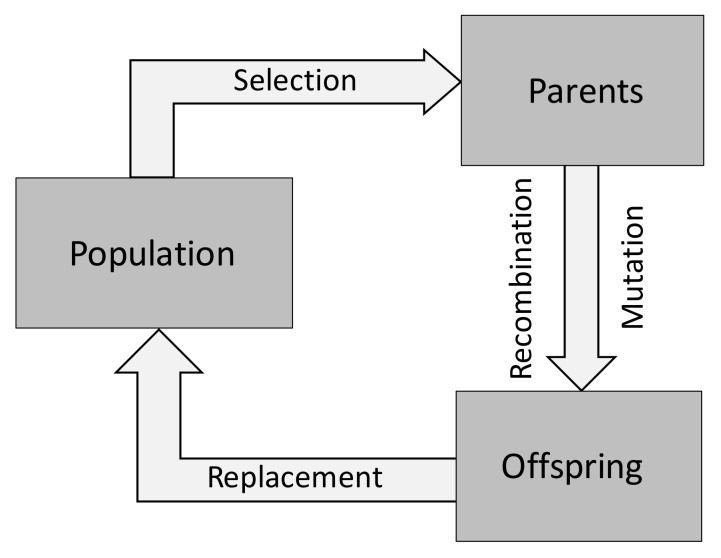
The evolutionary cycle.

The better known and more widely used multi-objective optimization algorithms are based on Evolutionary Algorithms (EA). These are meta-heuristics that mimic the process of natural evolution of a population of individuals, i.e., the solutions, along successive generations. They comprise Genetic Algorithms (GA), Evolutionary Programming (EP), Evolution Strategies (ES), and Genetic Programming (GP). Figure 4 illustrates schematically how this type of algorithms works. The individuals with higher performance in the environment will have more capacity to survive, which implies that they also have more capacity to be reproduced in the next generations. As in natural evolution, the offspring (new solutions) are generated by genetic operators such as crossover and mutation, inheriting most of the parent characteristics. The population of individuals, which are the potential solutions to the problem under study, evolves using the mechanisms of selection and variation. The selection operators enable the best individuals to have higher probability of being selected for generating offspring, and the variation operators allow the generation of new individuals [18,19,22]. Selection is based on the quality of each individual, which is given by a fitness function that is associated with the objective or objective functions for single or multi-objective optimization, respectively.

Based on the advantage of working with a population of solutions, multi-objective procedures were developed whereby the solutions evolve towards the optimal Pareto front in a single run. They are usually known as Multi-Objective Evolutionary Algorithms (MOEAs). In order to spread the population of solutions along the entire Pareto front, an additional operator measuring diversity is considered. The performance of the algorithm will depend on the balance between convergence, given by the value of the objective functions, and diversity, a measure of the distance between the solutions on the search space. Convergence and diversity are combined into a single fitness operator that is responsible for the selection of the solutions to be reproduced for the subsequent generations. This can be done in three ways: (i) based on Pareto dominance [22,23], (ii) scalarizing [24], and (iii) using indicator algorithms [25,26,27]. The research on MOEAs allowed the development of other types of multi-objective algorithms, such as Ant Colony Optimization (ACO) [28], Particle Swarm Optimization (PSO) [29], Simulated Annealing (SA) [30], and Differential Evolution (DE) [31].

**Figure 5:**
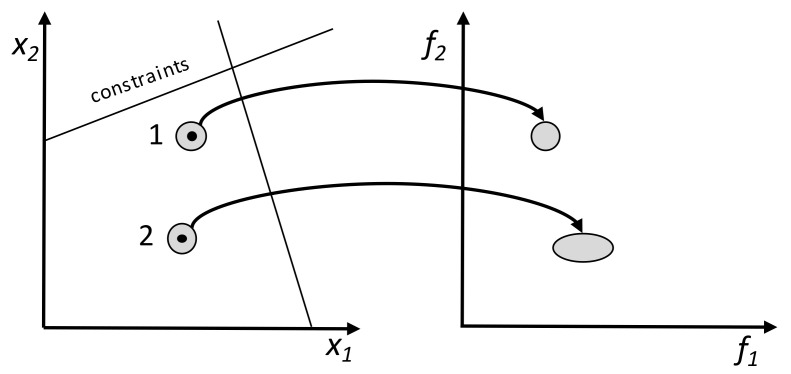
Concept of robustness in multi-objective environment. Solution 1 is more robust than Solution 2, as the same variation in the decision variables domain (*x*_1_,*x*_2_) produces less variation in the objectives’ domain (*f*_1_,*f*_2_).

Another important optimization topic concerns the robustness of the solutions obtained, i.e., the capacity of the solutions found of being robust against, for example, changes in the design variables. This may signify that the best solutions are not the ones selected, but, instead, other solutions that perform well for different ranges of the design variables. Robustness can be taken into account through expectation measures, which quantify simultaneously fitness and robustness, or by variance measures, which assess the deviation of the original fitness in the neighbourhood of the solution. Several combinations of expectation and variance measures have been linked to an MOEA, and applied to a few multi-objective problems, in order to select the most performing approach [32]. Gaspar-Cunha et al. [33] developed a set of benchmark problems to account for diverse types of robustness circumstances. The methodology was also assessed through application to some real problems. Figure 5 explains the concept in a multi-objective environment: solution 1 is more robust than Solution 2, as the same variation in the decision variables domain produces less variation in the objectives’ domain.

Since the result of a MOEA is a set of solutions, the decision maker is always challenged with the need to select the best (single) solution from this Pareto set. For this purpose, it is necessary to introduce, at some point of the optimization process, the preferences of one (or more) decision maker(s). A methodology based on a weighted stress function method was used for fitness assignment in MOEA [34]. This approach provides a fast convergence and a better final approximation performance, as measured by the usual quality indicators, when compared with traditional methods, such as aggregation functions.

## 4. Optimization Algorithms in Polymer Processing

### 4.1. Methodology

**Figure 6:**
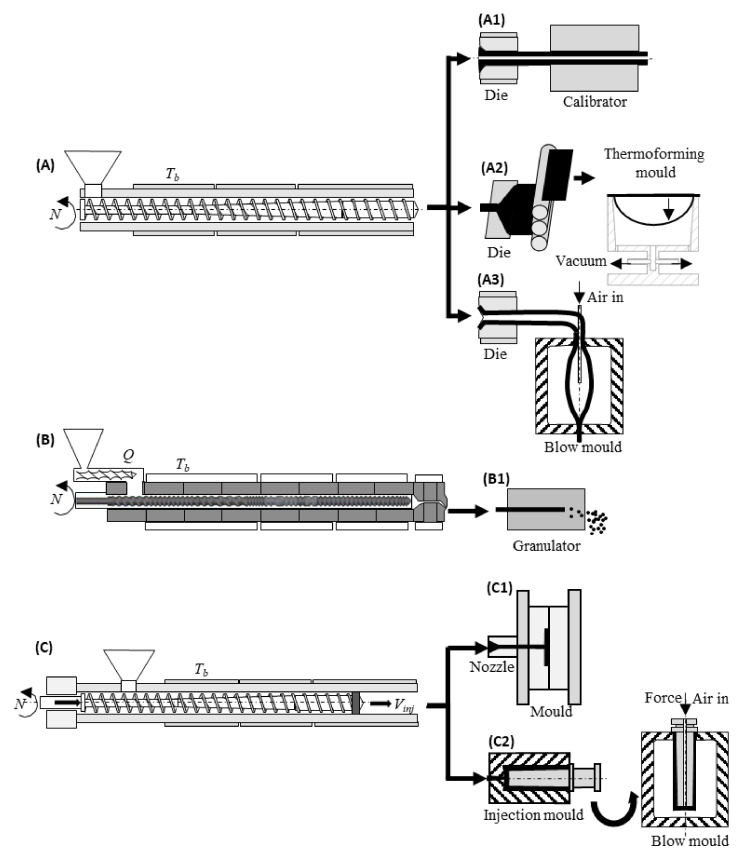
Polymer processing sequences targeted by the present review. (**A**) Single-screw extrusion of profiles (**A1**), flat film/sheet for thermoforming; (**A2**), extrusion blow moulding (**A3**); (**B**) co-rotating twin-screw compounding and pelletizing (**B1**); (**C**) injection moulding: (**C1**) mould (**C2**); injection blow moulding. Left: plasticating units; Right: shaping and cooling.

Figure 6 displays the polymer processing sequences targeted by the present review, as they can entail optimization problems. They include single-screw (Figure 6A) and twin-screw (Figure 6B) extrusion, injection moulding (Figure 6C), blow moulding (either based on extrusion (Figure 6A3) or on injection moulding (Figure 6C2)), and thermoforming (Figure 6A2), which produces 3D shapes from previously extruded sheets. Typically, these processes involve a plasticization step (carried out by the plasticating unit—which is an extruder in the case of Figure 6A and Figure 6B), encompassing material feed, melting, mixing, pressure generation, and pumping, followed by shaping and cooling. All these stages can be approached as optimization problems. In the case of the plasticating unit, it may be necessary to define the screw profile or set the operating conditions for a given polymer system/product combination. When extruding profiles (Figure 6A1), the design of the extrusion die and calibrator can be approached as optimization problems. Indeed, die design aims at defining the geometry of the flow channel that assures uniform melt velocity (and, if possible, also equal residence time) across the entire extrudate cross-section at the die exit. Similarly, the extrusion of flat film/sheet (Figure 6A2) with uniform thickness (or a pre-defined thickness variation) along its width requires a proper design of the die. Intermeshing co-rotating twin-screw extruders are extensively used in compounding and reactive extrusion operations (Figure 6B), the outcome of the process consisting of a new material in pellet form, to be subsequently converted into a final product by one of the available processing techniques. Since the geometry of these machines must be adapted to the requirements of each production, which may vary significantly, the screws and barrel are often built as assemblies of individual elements (which are supplied with different conveying, distributive and dispersive mixing abilities). Thus, screw design consists in selecting a given number of screw elements from a larger set of possibilities, and positioning them in the right sequence. This constitutes an interesting, albeit complex, optimization problem. In injection moulding (Figure 6C), mould design (Figure 6C1), screw design, and setting the operating conditions are well-recognized optimization problems. Finally, blow moulding also entails optimization challenges, as discussed in Section 2.

The discussion of the efforts reported in the open literature to solve the above optimization problems will be performed, whenever possible, using the following type of data (and respective acronyms):

1. Objective function. It can be Single Objective (SO), Aggregated Product (AP), Aggregated Sum (AS), or Multi-Objective (MO).
2. Optimization algorithm, e.g., Empirical, Regression, Direct, Gradient, Augmented Lagrangian (AL), Pattern Search (PS), Expert System (ES), Evolutionary Algorithm (EA), Differential Evolution (DE), Ant Colony Optimization (ACO), Stochastic Local Search (SLS), or Two-Phase Local Search (TPLS).
3. Modelling approach: unidimensional (1D), two-dimensional (2D) and three-dimensional (3D), using Analytical (A), Finite Differences (FD), Finite Volumes (FV) or Finite Elements (FE) approaches; whenever relevant, the actual software used is identified.
4. Decision variables, i.e., parameters to optimize. The aim can be to define the Operating Conditions (OC), Screw Design (SD), Screw Configuration (SC) (the last two will be explained below), or Die Geometry (DG). The number of variables considered in the problem is indicated between brackets in the tables below.
5. Other characteristics, related with the process/modelling, the optimization, or others.

### 4.2. Single-Screw Extrusion

This section reviews the previous optimization studies of the plasticating unit of single-screw extruders (SSE). The decision variables that have been considered are related to the optimization of the operating conditions (screw speed and barrel temperature profile) and/or of the screw geometry. Conventional and barrier screws, as well as units with a grooved barrel in the feed zone have been studied. Table 1 summarizes the features of the various publications found in the literature.

The earliest attempts to optimize single-screw extrusion used statistics, regression, and response surface analyses based on experimental data [35] and computer modelling [36,37,38]. Helmy and Parnaby [38] applied a steady-state hill-climbing optimization method, together with an analytical modelling routine, to design screws, making this a good example of the application of traditional optimization methods (see Figure 3A). They implemented an iterative procedure where the required pressure and flow rate at the die, and the constraints (e.g., machine dimensions, screw strength and product quality), are initially defined. The search is made considering a single objective (screw power efficiency). Potente et al. [39] used an 1D analytical modelling software to optimize a screw for a grooved barrel extruder, using a trial-and-error procedure. Wortberg et al. [40] employed the same modelling software to develop an expert system to optimize extrusion but recognized the necessary intense interaction between process simulation and expert system, in order to create a database with an adequate dimension, capable of working with variations in operating conditions, material properties and system geometry.

**Table 1:** Previous publications on the optimization of single-screw extruders.

Objective Function	Optimization Algorithm	Modelling Approach	Decision Variables	Other Characteristics	Authors (Year) Reference
SO	Direct	1D-A	SD	Step-by-step	Helmy and Parnaby (1976) [38]
SO	Empirical	1D-A	SD	Grooves	Potente et al. (1992) [39]
SO	ES	1D-A	OC + SD		Worteberg et al. (1994) [40]
SO	Empirical	1D-A	SD	Step-by-step	Chung (1998, 2016) [8,41]
SO	Empirical	1D-A	SD	Zone-by-zone	Rauwendaal (1986) [42]
SO	AL	3D-N	SD		Altinkaynak (2010) [43]
AP	Empirical	1D-A	OC		Potente et al. (1993, 1994, 1996) [44,45,46]
AP	Regression	1D-A	SD	Statistical	Potente and Zelleröhr (1997) [47]
AP	Regression	1D-A	SD	DOE	Potent and Krell (1997) [48]
AP(3)	Regression	1D-A	OC(2) + SD(1)		Wilczyński et al. (2001, 2003) [49,50]
AP(3)	Regression	1D-A	OC(2) + SD(1)		Wilczyński et al. (2004) [51]
AS(3)	Regression	1D-A	SD		Thibodeau and Lafleur (2000) [52,53]
AS(2)	EA	1D-A	OC(2) + SD(1)		Nastaj and Wilczyński (2018) [54]
AS(2)	EA	1D-A	OC(2) + SD(1)	Starve-feed	Nastaj and Wilczyński (2020) [55]
AS(2)	DE + PS	Experimental	OC(1)	Various techniques	Abeykoon et al. (2011) [56]
AS(4)	EA	2D-N	OC(4)		Gaspar-Cunha et al. (1998) [57]
AS(4) + MO(4)	EA	2D-N	OC(4)		Covas et al. (1999) [58]
MO(7)	EA	2D-N	SD(6)		Gaspar-Cunha et al. (2001) [59]
MO(5)	EA	2D-N	SD(5)	Barrier screws	Covas et al. (2004) [60]
MO(2)	EA	2D-N	OC(4) + SD(6)	Mixing	Domingues at al. (2012) [61]
MO(5)	EA	2D-N	SD(4)	Barrier screws	Gaspar-Cunha et al. (2006) [62]
MO(19)	EA	2D-N	OC(3)	Scale-up	Covas and Gaspar-Cunha (2009) [63]
MO(9)	EA	2D-N	SD(4)	Scale-up	Gaspar-Cunha and Covas (2014) [64]
MO(3)	EA	2D-N	SD(4)	Robustness + DM	Denysiuk et al. (2018) [65]
MO(5)	EA	2D-N	OC(4) + SD86)	Innovization	Deb et al. (2014) [66]

Some authors claimed to design screws “scientifically”. Chung [8,41] proposed designing the entire screw through a sequence of steps aiming to match a given output. From a balance between heat conducted and heat generated by viscous dissipation, the depths of the metering and feed sections were defined. Then, some adjustments were made taking into account practical/empirical rules. Rauwendaal [42] defined the screw geometry by solving the analytical equations pertaining to each functional screw zone in order to meet the relevant objectives, such as power consumption and output.

Traditional (and inefficient) methods are still being used to optimize SSE. For example, Altinkaynak [43] developed a full 3D finite element code for melting and metering zones, which was used to optimize the screw pitch and depth of the metering zone. However, since the Augmented Lagrangian method (an algorithm for solving constrained optimization problems) was adopted to maximize output, together with a constraint (the melt temperature at the extruder outlet could not exceed a pre-defined limit), the relevance of the results obtained was very limited. Hence, this work illustrates the need to find a good balance between the use of computationally demanding modelling codes and the optimization method.

Potente et al. [44,45,46,47,48] reported one of the first systematic attempts to consider multiple objectives by means of a simple scalar objective function, together with a 1D analytical modelling program. For instance, Potente et al. [44,45,46] assumed the M-square root (M is the number of objectives) of the product of the individual objectives (such as output and length of screw required for melting) to avoid any of them assuming a zero value. However, an empirical procedure was employed to optimize the process. Potente and Zelleröhr [47] optimized the process with a statistical method and a regression analysis, obtaining contour plots for the quality function from a regression analysis to the results generated by the modelling software. Potente and Krell [48] proposed a methodology for screw design involving a DOE (Design of Experiments) and multiple regression. A similar strategy using the STATISTICA software and a 1D analytical process description was adopted by Wilczyński et al. [49,50]. The barrel temperature, screw speed and screw channel depth in the metering zone were optimized (using a M-square root of the product of the normalized objectives) in order to maximize mass flow rate and minimize power consumption and melt temperature at the die outlet. Subsequently [51], these results were compared with those obtained using Artificial Neural Networks (ANN) instead of the statistical analysis. It was concluded that the statistical strategy produced better results. Thibodeau and Lafleur [52,53] adopted a five-level central composite statistical model to design screws that maximize mixing and minimize melt temperature in the feed zone through a desirability function. The optimum was found on a response surface determined by a 1D analytical modelling program.

Nastaj and Wilczyński [54] applied EAs to optimize the screw speed and the length of the metering zone that maximized output and minimized mechanical power consumption, with a weighted sum as global objective function. Similarly, the same authors [55] optimized starve-fed/flood-fed single-screw extruders in terms of screw speed, barrel temperature, and length of the metering zone, for the same two objectives. They concluded that starve-fed extrusion performed better.

Abeykoon et al. [56] compared the performance of differential evolution and particle swarm algorithms, both based on the use of a population of solutions. The aim was to find the barrel set temperatures that minimized the difference between the average melt temperature at the die and the temperature required by the process, as well as the temperature variance at the die. These two objectives were aggregated in a single objective function through a weighted sum. The melt temperature was evaluated using a static nonlinear polynomial model whose parameters were obtained from experimental data. Data analysis allowed them to build a model for evaluating the objectives, while the population-based algorithms were used for the optimization.

**Figure 7:**
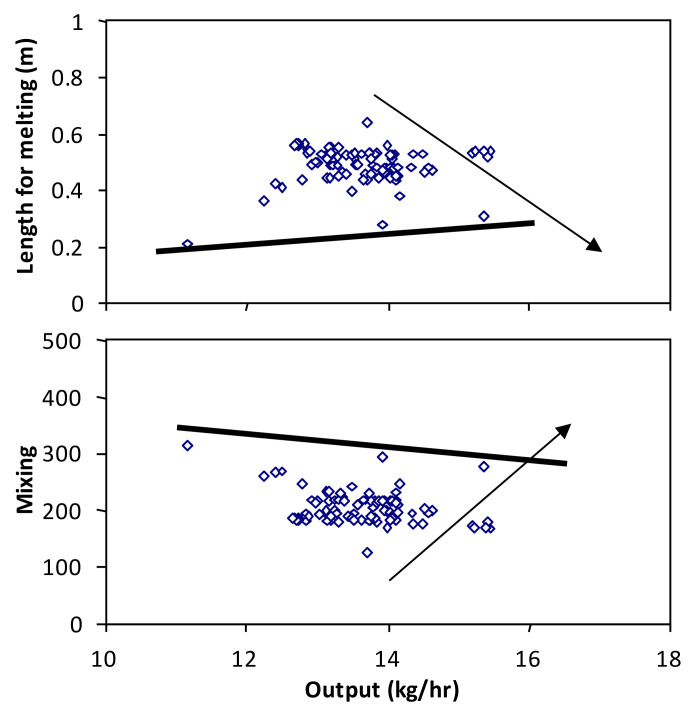
Pareto curves after optimization of the operating conditions of an SSE in order to maximize output and mixing, and minimize the length of screw required for melting.

Gaspar-Cunha et al. [57] analysed the advantages and shortcomings of implementing an optimization methodology based on the interplay between a modelling package, an objective function, and an EA to solve single-screw extrusion problems. The various objectives were taken into account through an aggregation function (the weighted sum) to define the operating conditions (screw speed and barrel temperature profile) that produced the desired output and/or product characteristics. Although the approach was able to find solutions with physical meaning, changing the weights of the aggregation function did not allow to access most of the solutions along the Pareto front, as the algorithm converged to the extremes of the search space. Therefore, multi-objective algorithms seemed a better alternative. Indeed, upon applying an MO approach based on EAs to the same SSE problem, the trade-off between four different objectives was established, providing a better understanding of the features of the extrusion system under study [58]. Figure 7 shows the two-dimensional Pareto fronts after optimizing the operating conditions of an SSE in order to maximize output and mixing, and minimize the length of screw required for material melting. The aim was to approach the edges, as indicated by the arrows. The best points are those near the lines in each graph. Nevertheless, during the optimization process, it was found that when more than two objectives were considered, most of the solutions were non-dominated. Thus, a new MOEA based on a clustering strategy was developed to select the solutions while maintaining the diversity along the Pareto front. The same MOEA was used to optimize the screw geometry (described by six parameters) for the same SSE problem [59], the methodology being sensitive to changes in the design parameters but obviously dependent on the ability of the modelling routine to provide precise predictions. The approach was extended to the design of Maillefer barrier screws, assuming five design screw parameters and five objectives [60]. Domingues et al. [61] modelled the evolution of the morphology of immiscible liquid–liquid and solid–liquid systems in SSE and used these data to compute distributive and dispersive mixing indices. These were added as new objective functions to optimize the operating conditions and screw geometry using the same MOEA. Subsequently, a modified strategy was adopted to optimize altogether conventional and Maillefer barrier screws [62]. A structured chromosome representation was adopted, in which various parts of the chromosome represented the same variables and the genotype representation was based on a hierarchy (i.e., one of the decision variables is a flag indicating the type of screw). The results showed that different regions of the Pareto front were attained by the two screw types.

Scale-up in SSE often consists in finding the geometry and/or the operating conditions of a target extruder (in general, of industrial production size) in order to obtain materials or products with the same characteristics of those developed with existing equipment (commonly, a laboratory extruder). This should require that flow and heat transfer at the two scales are similar, but the problem is difficult to solve as changes in scale affect differently the various process parameters. Covas and Gaspar-Cunha [63] approached scale-up as a multi-objective optimization problem, where the aim was to minimize the differences in performance between extruders of different sizes while simultaneously satisfying several objectives. Screw speed and barrel temperatures in three zones were initially assumed as decision variables. Subsequently, the same methodology was applied to screw design, considering four-screw geometrical parameters and nine objectives [64].

Robustness and decision-making strategies have also been included in the solution to SSE problems [65]. In this way, it becomes possible to focus the search on solutions that converge to regions where the preference was defined either by the relative importance of the objectives or by considering the robustness of solutions against perturbations in the design variables. Deb et al. [66] proposed an “innovization” methodology to capture relationships between the relevant process parameters (i.e., the decision variables) and the objectives from the final results of a multi-objective optimization algorithm. After applying a MOEA, the optimal trade-off solutions are analysed and the interactions between the parameters are obtained automatically. The procedure was applied to an SSE problem, a set of rules relating the relevant decision variables with the objectives for each case studied being established.

### 4.3. Twin-Screw Extrusion

**Figure 8:**
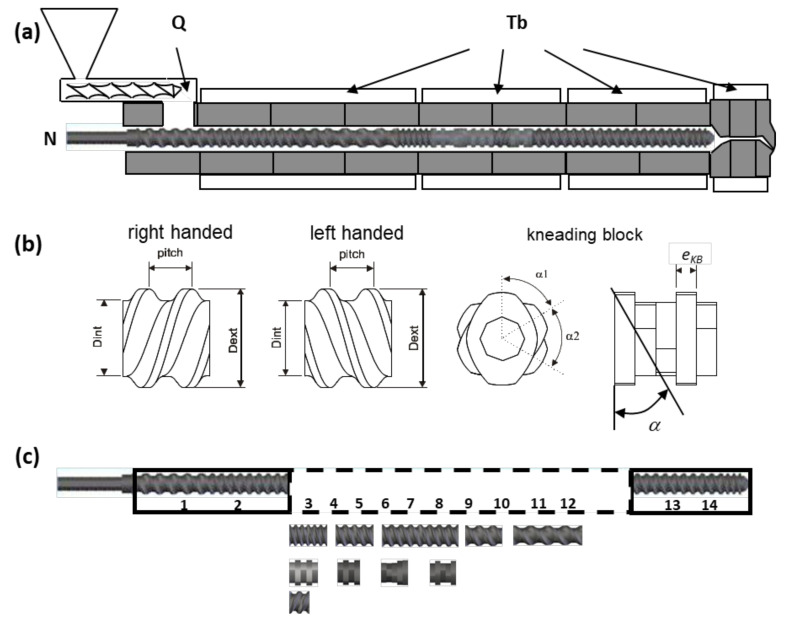
Optimization of co-rotating twin-screw extruders (TSE): (**a**) operating conditions—screw speed (N), feed rate (Q) and barrel and die set temperatures (Tb); (**b**) geometry of individual screw elements; (**c**) position of a set of individual screw elements (5 conveying elements, 3 kneading blocks, and 1 left-handed element) along the screw shaft.

As illustrated in Figure 8, optimization of twin-screw extruders (TSE) may involve: (i) the definition of the operating conditions (*N*-screw speed, *Q*-feed-rate, and *T_b_*-barrel temperature profile); (ii) the determination of the geometry of individual screw elements; and/or (iii) the determination of the screw configuration, (i.e., finding the best location along the screw axis of existing screw elements). These problems arise within the context of compounding, reactive extrusion, extrusion, or scale-up and can involve co-rotating or counter-rotating intermeshing twin-screw extruders. Table 2 summarizes the features of the previous studies on these topics.

As with SSE, co-rotating twin-screw extruders were initially optimized using empirical approaches based on experimental and computational data. Potente et al. [67,68] developed SIGMA, a 1D modelling software, but details on the optimization method, including design variables and objectives, were not given. The results achieved seem to have been based on a trial-and-error procedure. Vainio et al. [69] investigated experimentally different screw profiles for the preparation of an uncompatibilized immiscible PA6/PP blend with the aim of optimizing the screw configuration. Although the process parameters influencing mixing were identified, no systematic optimization method was offered. Berzin et al. [70] graphically optimized the cationization of wheat starch in a laboratory extruder in terms of screw configuration and operating conditions, taking into account geometrical and process constraints, with the aim of scaling-up the results to a larger industrial extruder. Five different screw configurations were analysed, assuming the minimization of the specific mechanical energy and the maximization of the reaction efficiency. The scale-up procedure used the same type of data for screws geometrically similar to those studied for the smaller extruder.

Various authors adopted statistical methods for TSE optimization. The analysis applied by Maridass and Gupta [71] was based on experimental data on the recycling of natural rubber vulcanizates in a counter-rotating twin-screw extruder. The aim was to find the barrel temperature and screw speed that maximized the properties of the material obtained. For that purpose, the contour plots obtained were analysed visually. Ulitzsch et al. [72] selected a response surface methodology to optimize the synthesis of vinyltrimethoxysilane-grafted ethylene–octene–copolymer using experimental data. Five process parameters and their interactions were optimized, in order to maximize both the grafting degree and efficiency. Second-order interaction effects existed, making this process difficult to control with such a simple optimization methodology. Fukuda et al. [73] used a DOE to analyse separately two different scale-up rules, one based on the volumetric flow rate, the other related to dispersive mixing, with the aim of optimizing the operating conditions of the target extruder.

Potente and Thümen [74] applied a gradient method to optimize the radial and flight clearances of conveying screw elements in order to maximize the pressure gradient and minimize the temperature gradient and power consumption.

Zhang et al. [75] employed a single-objective EA to optimize free radical bulk polymerization, with the aim of defining the barrel temperature and screw length that maximized both monomer conversion and monomer conversion per unit energy consumption. A weighted sum took into account the two objectives. A weighted sum and a MOEA were adopted to set the operating conditions of three extruders with different screw configurations [76] while using the Ludovic software [77] to evaluate the objective functions.

When optimizing operating conditions, or the geometry of individual screw elements, the characteristics of these tasks are identical to those of equivalent SSE problems, since the decision variables to optimize vary continuously in the search space. However, defining the best location of screw elements along the screw shaft is a discrete and combinatory problem. Gaspar-Cunha et al. [78] used a modified MOEA to define the most adequate location of a pre-selected set of screw elements (denoted as screw configuration problem, SC). An analogy was made with the travelling salesman problem, or sequencing problem in operations research, with the cities being the screw elements that must be covered sequentially by the traveling salesman in order to maximize a prescribed performance. Within the obvious limitations of the exercise, the results were validated experimentally, and the methodology was also applied to ∑-caprolactam polymerization via reactive extrusion. Later, the robustness of the solutions, considering changes in the value of the decision variables, was also taken into account [60]. An alternative approach using Stochastic Local Search (SLS) algorithms to tackle the SC problem was also attempted [79]. An efficient single-objective iterative improvement strategy, based on various neighbourhood structures, neighbourhood search strategies, and neighbourhood restrictions, was proposed, whereby the algorithms were embedded into a variation of a bi-objective two-phase local search (TPLS) framework. The results were compared to those obtained by the previous MOEA, evidencing a higher-quality approximation to the Pareto front, with a faster convergence. Process modelling consisted of a global plasticating treatment of co-rotating twin-screw extrusion [80]. Given the good results obtained, Teixeira et al. [81] solved the SC problem through the hybridization of different local search procedures, including Pareto local search and TPLS algorithms, with two different population-based algorithms, a MOEA and a Multi-Objective Ant Colony (MOACO). This approach outperformed the other algorithms studied and their combinations. With the aim of exploring the full potential of the hybrid algorithm, the influence of the MOACO algorithm parameters was investigated, and the results obtained were compared with those of MOEA and TPLS algorithms [82]. It was concluded that the hybridization of the MOACO algorithm has a significant potential for solving the SC problem.

Teixeira et al. [83] adopted a MOEA algorithm to solve the SC problem for starch cationization by reactive extrusion, aiming at the minimization of the specific mechanical energy and the maximization of output and reaction conversion. As far as scale-up in TSE is concerned, Gaspar-Cunha and Covas [84] employed a multi-objective optimization strategy to define the geometry (location of eight screw elements) and operating conditions of the target extruder that minimized the differences in viscous dissipation, specific mechanical energy, and average strain between the target and reference machines.

**Table 2:** Previous publications on the optimization of twin-screw extruders.

Objective Function	Optimization Algorithm	Modelling Approach	Decision Variables	Other Characteristics	Authors (Year) Reference
Not defined	Empirical	1D-A	Not defined		Potente et al. (1994, 1999) [67,68]
SO	Empirical	Experimental	Not-defined	Mixing	Vainio et al. (1995) [69]
SO(2)	Regression	1D-Ludovic	OC(3) + SD(1)	Reactive Extrusion	Berzin et al. (2007) [70]
SO	Regression	Experimental	OC(2)	Counter-rotating	Maridass and Gupta (2004) [71]
SO(2)	Regression	Experimental	OC	Reactive Extrusion	Ulitzsh et al. (2020) [72]
SO(2)	Regression	Experimental	OC(2)	Scale-up	Fukuda et al. (2015) [73]
AP(3)	Gradient	1D-A	SD(2)	Conv. elements	Potente and Thümen (2006) [74]
AS(2)	EA	2D-numerical	OC(1) + SD(1)	Reactive Extrusion	Zhang et al. (2015) [75]
AS + MO(6)	EA	1D-Ludovic	OC(4)		Gaspar-Cunha et al. (2002) [76]
MO(7+2)	EA	1D-Ludovic	OC(4) + SC(10)	Reactive Extrusion	Gaspar-Cunha et al. (2005) [78]
MO(5)(7)	EA	1D-Ludovic	SD(4) + SC(10)	Robustness	Covas et al. (2004) [60]
MO(3)	SLS	2D-FD	SC(14)		Teixeira et al. (2011) [79]
MO(3)	EA + ACO + SLS + TPLS	2D-FD	SC(14)		Teixeira et al. (2012) [81]
MO(3)	ACO + TPLS	2D-FD	SC(14)		Teixeira et al. (2014) [82]
MO(3)	EA	1D-Ludovic	OC(1) + SC(14)	Reactive Extrusion	Teixeira et al. (2011) [83]
MO(3)	EA	2D-FD	SD(1) + SC(8)	Scale-up	Gaspar-Cunha and Covas (2011) [84]

### 4.4. Dies and Calibrators

Extrusion dies aim at converting the circular flow at the outlet of the extruder into a flow with a specific cross-section (to produce film/sheet, pipes, profiles, etc). The latter may not correspond to the actual product cross-section/dimensions, as the die geometry must compensate for all the shape/dimensional changes of the extrudate along the extrusion line. As a matter of fact, due to its viscoelastic nature, the extrudate will swell progressively as it leaves the die, but this might be partially/totally offset by the draw down created by the haul-off. During cooling, thickness differences may arise in the cross-section due to gravity flow, and the extrudate will shrink and might distort due to buoyancy forces in a water tank. Thus, not only are extrusion dies built in such a way that they attempt to anticipate subsequent changes in the shape of the extrudate, but they also allow for some local adjustments in the channel geometry. In addition, whenever possible, the external contour of the extrudate is corrected prior to cooling by means of a calibrator.

Extrusion dies usually comprise an adapter, which converts the circular flow from the extruder into the required channel shape for extrusion, and a parallel zone with constant cross-section, which allows for some macromolecular relaxation. The ensemble adapter/parallel zone should be designed in such a way that the velocity and residence time of all the individual melt streams in the cross-section are uniform. To ensure this, and despite of the wide variety of extruded shapes, there are generally three approaches to designing the adapter:

(i) Using a manifold, i.e., use a larger channel upstream to distribute the flow transversally, prior to its progress downstream. The die geometry is such that a central flow stream has a shorter path in the manifold and a longer path in the shallower parallel zone, while the reverse occurs for a flow stream near to the edges. This approach is frequently adopted for the production of cast film and sheet, wire insulation, and in extrusion blow moulding.
(ii) Using a cylindrical mandrel to convert the circular flow from the extruder into an annular flow. Since the classical torpedo-type solution with its supports (known as spider legs) creates unbalanced flow and strong weld lines, it was progressively replaced by basket-type dies and spiral mandrel dies. The mandrels of the latter are designed in such a way that the flow from the extruder is divided into individual melts that feed helical channels with decreasing depth along their length in the mandrel. Thus, the helical flow is gradually converted into an axial annular flow.
(iii) Change gradually from the inlet circular channel into the desired cross-section. The design of dies for hollow profiles, or for profiles containing thickness differences in their cross-section is particularly challenging.

Rakos and Sebastian [85] proposed an empirical optimization procedure to define the geometry of different types of dies using a numerical modelling code, but no details were given concerning the objectives and design variables. In the following sections, each type of die is studied separately.

#### 4.4.1. Manifold Dies

Matsubara [86,87] solved the analytical flow modelling equations in order to determine a major design variable (the variation along the length of the manifold radius) of a coat hanger die that assured uniform flow rate and residence time across the width, and extended the methodology to T-dies [88,89]. Table 3 shows that the design of manifold dies as an optimization problem was carried out using a single objective function and that optimization procedures included empirical methods, regression analyses, sequential quadratic programming, gradient techniques, or evolutionary algorithms.

Winter and Fritz [90] recommended the use of a specific design procedure for a coat hanger die, taking into account uniformity of the exit velocity and average residence time, regardless of flow rate or polymer viscosity, and considering flow separation. The geometry of dies with a square and circular manifold and constant thickness of the parallel section was defined, in which the design variables were the width or the diameter of the manifold as a function of its length and the height of the parallel section.

Liu et al. [91] developed a method to optimize coat hanger dies with non-circular manifold, to avoid dead spots in the transition to the parallel zone. The aim was to define the geometries of the manifold and parallel zone that delivered uniform flow, while keeping identical residential time distribution for different polymers and operating conditions. A numerical modelling program was used to evaluate the solutions. The same empirical strategy was used by Lee and Liu [92] to design a coat-hanger die with a linearly tapered inner cavity and a straight outer cavity, but taking into account inertial, gravitational, and viscous effects. Later, Liu et al. [93] and Yu and Liu [94] proposed a unified lubrication approximation to model the polymer flow inside the die with the aim of designing the same coat-hanger die [93] and a tapered coat-hanger die [94].

Na and Kim [95] applied an empirical approach to design linearly tapered coat-hanger dies with circular manifold (in terms of slot thickness, manifold angle, and land length) with the aim of obtaining uniform flow rate distribution at the die exit in the transverse direction, using a 3D finite element modelling code. Even recently, a simple empirical approach was used by Huang et al. [96] to design coat hanger dies that maximize the uniformity of the velocity distribution at the die exit, defining as decision variables the manifold radius and angle and the slit height.

The application of regression techniques based, for example, on Taguchi methods, enabled the development of more systematic optimization approaches. This is the case for Chen et al. [97], who investigated the effect of material rheology, gap thickness, manifold angle, and flow rate on the thickness uniformity of coat hanger dies. The Taguchi method was used to optimize the geometry of dies with different widths, the solutions being evaluated with an analytical model, and flow rate was also appended as design variable. Recently, Razeghiyadaki et al. [98,99] used a response surface method to optimize the geometry of a coat hanger die in order to obtain uniform velocity at the die lips. The response surface was generated from computations using a commercial package, and a central composite DOE defined the conditions for the calculations. A spline curve was used to outline the geometry using five variables (depth and the width of the die and three nodes of the spline). The optimization involved minimizing the quadratic function obtained. Lebaal et al. [100] determined the geometry of a coat-hanger die using a global response surface method with Kriging interpolation (a regression technique), and Sequential Quadratic Programming (SQP) to minimize the global difference between the local velocities at the die exit and the average value, assuming as a decision variable the depth of the distribution channel and as a restriction the pressure required by the flow. Later, four decision variables were considered, namely the depth and the opening of the channel repartition, the gap, and the height of the relaxation zone [101]. The same methodology was applied to define the depth of the distribution channel and the operating conditions [102], as well as to design a wire coat hanger based on the same flow balance principle [103]. It is important to note that SQP requires that the objective function and the constraints are twice differentiable, which was possible in this case because the objective function and the constraints were defined by regression prior to the application of SQP.

Smith et al. [104] proposed a systematic optimization methodology combining process modelling, a design sensitivity analysis (using both direct and adjoint methods), and optimization based on a gradient technique. The aim was to keep the non-uniformity of the velocity profile across the die exit below a certain level and to minimize pressure drop, i.e., to achieve product homogeneity with minimal processing cost. The design variables were the length of the parallel zone and the cross section of the flow channel. The residence time in the die was considered subsequently [105]. Then, with the aim of minimizing the die length and satisfying constraints related to uniform residence time and exit velocity, the inlet pressure, the manifold height, and the shape of the parallel zone were taken as variables [106]. Furthermore, to determine the geometry of a sheet die that would best accommodate a range of operating conditions, Smith [107] optimized 811 half-height design variables that described the die cavity thickness distribution for an example where the inlet and outlet die half-heights were fixed. Sun and Gupta [108] applied a gradient quadratic penalty method to define the geometry of a coat hanger die (using nine decision variables) that minimized the velocity variation across the die exit without excessively increasing the pressure drop. The penalty method consisted in adding a burden to the objective function for surpassing a pressure drop threshold. Bates et al. [109] applied a gradient optimization method to determine the optimum geometrical profiles of a restrictor (choker bar) necessary to obtain a uniform flow distribution of a slit die to be used in a range of applications involving three materials with varying degrees of shear thinning, each at a high and a low flow rate. The restrictor was optimized considering the height of five points along half the width of the die. Later, this study was extended to include regression and EA optimization algorithms [110].

A single objective EA was used by Michaeli and Kaul [111] to optimize a T-shaped manifold in order to minimize the standard deviation of the local velocities at the die exit, the decision variables being a few points in the mesh that defined the flow path. The same optimization strategy was applied to define the geometry of a coat hanger die, taking as design variables two parameters related with the radius of the manifold and the height of the parallel zone [112]. While also using a single objective EA, Sun and Wang [113] optimized four geometrical parameters of the manifold. The aim was to minimize the stagnation temperature (a combination of static and kinetic temperatures), and the solutions were evaluated using a 3D numerical commercial modelling code. Meng et al. [114,115] designed a double coat hanger die with a manifold of quadratic geometry in order to distribute uniformly the melt across a large width. A single-objective EA was selected to define the gap and the manifold angle.

Several authors recognized the need to take in several objectives, hence using aggregation functions (e.g., weighted sum) together with single objective optimization algorithms. Han and Wang [116] applied a regression technique (based on an orthogonal array design) to determine the manifold angle and gap height of a coat-hanger die that minimized the variation of the outlet velocity and the residence time, using a 3D numerical modelling code. The same optimization methodology (see also [104]) was used to include the effect of the variability of the set temperature or of material properties, i.e., a robustness analysis. In this case, the objective function was the weight sum of the inlet pressures for each flow condition that are induced by changes in temperature and/or material properties [117,118]. An arbitrary gap height distribution in the manifold and different polymer rheological models were also added [118]. Wang and Smith [119,120] solved the same problem using SQP. Zhang et al. [121] optimized the geometry and operating conditions of a coat hanger die using a single objective EA consisting of the weighted sum of three objectives (minimization of the mechanical deformation of the die, of the pressure drop, and of the variation of the outlet velocity). The solutions to be evaluated were obtained through the application of a DOE, from which a regression model was obtained to be used by the EA.

Only recently have MO optimization methods been applied to the definition of the die geometry. Lee et al. [122] evaluated the performance of different optimization strategies (comprising a design of experiments, the response surface model and two different MOEAs) for the delineation of the geometry of a coat hanger die. The latter was divided into sectors that resulted in three case studies involving the definition of three, eight, and twelve design variables, respectively. In parallel, two objectives were selected— minimization of the total pressure drop and maximization of a flow uniformity parameter—which were evaluated using a commercial 3D modelling software. Considering these two objectives simultaneously, Han and Wang [123] used an MOEA to optimize the same geometrical parameters, taking as starting point the geometry of a previously optimized die [116]. Later, the same authors [124] optimized a double coat-hanger die with a quadratic geometry manifold using identical optimization techniques (regression and MOEA).

#### 4.4.2. Mandrel Dies

Table 4 identifies the previous studies concerning the optimization of mandrel dies. Huang [125] proposed a strategy with two steps to optimize the geometry of a spiral mandrel die (that can be utilized for the extrusion of pipes and blown films). The Taguchi method was used to define a set of geometries able to assess the flow balance principle; then this set of geometries was evaluated iteratively taking into account total pressure drop, degree of mixing, and residence time distribution. It was concluded that the best solution to use in real practice results from a balance between the different objectives, and hence a multi-objective strategy must be pursued. Mu et al. [126] adopted a MOEA to optimize the geometry of an annular die, aiming at minimizing local differences in outlet velocity and minimizing the swell ratio while restricting the shear stress to a critical value that guaranteed steady extrusion. An ANN, trained with 3D numerical modelling results, was used to evaluate the solutions, while the decision variables were the channel contraction angle, the flow gap, and the relative length of the parallel zone.

#### 4.4.3. Profile Dies

Two main approaches (not necessarily exclusive) have been generally adopted for the design of profile dies (see Table 5 for a summary of the previous studies published): optimization of the flow balance at the die exit and correction of the shape/dimensions of the die exit for the effect of post-extrusion extrudate-swell (this is sometimes denoted as the Inverse Extrusion Problem (IEP) [127]. According to Pittman [128], the design of profile dies should include the following aspects: (i) consider as much as possible the thermomechanical phenomena, such as an appropriate rheological description and kinematics, eventual wall slip conditions, extrusion instabilities, material residence time and degradation, extrudate swell, and draw-down and thermal effects; (ii) select the best strategy, e.g., flow balancing, streamlined channels, avoiding-cross-flow, using flow separators, and designing for extrudate-swell; (iii) formulate clearly the optimization in terms of objective function, constraints, decision variables, algorithms, and optimization strategies; and (iv) perform a clear geometry and mesh parameterization.

Legat and Marchal [127] designed a square die by solving the IEP for the die channel shape given the extrudate geometry, based on an implicit formulation. Tran-Cong and Phan-Thien [129] proposed an empirical optimization method to take into account the effect of extrudate-swell on die design. Flow modelling used the boundary element method, and the free surface was modelled based on particle path lines that were optimized at every iteration. Also through a trial-and-error empirical approach, Hurez et al. [130] optimized the lengths of the die land channels using analytical flow modelling. Three empirical strategies based on cross flow minimization were utilized by Švábík et al. [131] to achieve flow balancing by varying the die land length. However, these methods are only able to deal with simple dies. With the aim of solving the IEP, Gifford [132] discussed the concept of target profile, i.e., the final profile to be obtained after the extrudate-swell, and how to deal with the free surface using surface particles that must fit the target profile. Rezaei et al. [133] optimized the length of the die lands of a profile die using an empirical scheme based on a sensitivity analysis in order to balance the flow at the die exit.

One of the first systematic optimization approaches was made by Coupez et al. [134], who adopted the simplex method to optimize the geometrical parameters of a profile die in terms of flow balancing, based on 3D numerical modelling but with unclear decision variables. Ready and Schaub [135] used of a regression optimization method based on a response surface methodology with the results obtained by an adaptive 3D numerical method and defining as decision variables the corner positions of macro-blocks located in the numerical mesh. An optimization approach based on the gradient-free method (specifically, bound optimization by quadratic approximation) was applied to manifold and profile dies [136]. The method uses a regression approach based on a quadratic response surface obtained from the modelling calculations. Spline lines approximate the domain boundary, with design variables corresponding to the weights and locations of the 22 control points that define the splines. The objective function was the variance of the local maximal velocity as compared to the average maximal velocity over all die sections. Based on the same optimization framework, Pauli et al. [137] considered that a good die design must also attain a homogeneous extrudate-swell across the die exit and thus applied the two objectives simultaneously to a U-shape profile using 19 × 9 control points.

**Figure 9:**
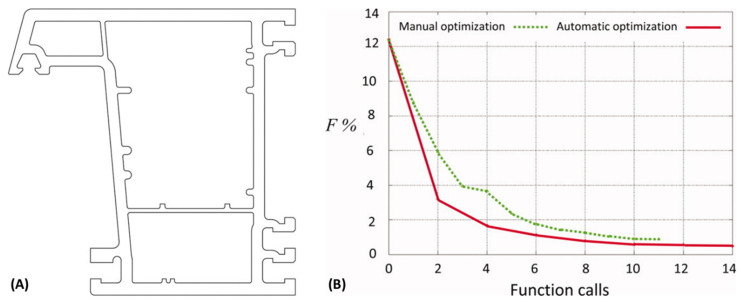
Optimization of a die for the production of a hollow profile: (**A**) required fixed-window profile cross section and (**B**) evolution of the objective function versus function call for automatic and manual optimization (adapted with permission from [144]).

Sienz et al. [138,139] applied a gradient optimization method based on a sensitivity analysis to design a flow balanced profile die. The same method was used to maximize the velocity at the die exit of another die using as design variable the land height for the inner branches, the solutions being evaluated by a 3D numerical commercial code, and the optimized results being assessed experimentally [140]. An expert-system-driven optimization was also employed [141]. Ettinger et al. [142] and Ettinger [143] proposed a methodology to design profile extrusion dies for Poly(vinyl chloride) (PVC) involving modelling, parameterization techniques, optimization strategies, and the determination of material parameters. Parametrization was based on key-points (KP) with two coordinates (x,y) and a radius (i.e., the design variables), while flow modelling was carried out with FE performed on 2D die cross-section slices. The optimization problem was solved employing a gradient optimization algorithm, but other strategies were also tested (global and sequential optimization schemes, height approximation method and parallel decoupled scheme), with a view to flow balancing. Later, the authors designed several complex window profiles with the aim of guaranteeing that the right quantity of material was delivered to all parts of the die exit. They fixed between 2 and 46 design variables and used the gradient optimization method based on the calculation of the sensitivities obtained from the rates of change of the objective function with respect to the decision variables [144]. Figure 9 illustrates the application of this methodology to design complex die geometries. Figure 9B compares the evolution of the objective function between manual and automatic optimization. As can be seen, the convergence of automatic optimization is attained after 10 function calls and that automatic optimization performs better than manual optimization.

Yilmaz et al. [145] determined the height of the thick channel and the length of the narrow channel of an L-shape profile die that would balance the flow, using simulated annealing together with a kriging meta-model to estimate the modelling results based on 3D numerical computations. Very recently, Spanjaards et al. [146] proposed solving the IEP using the theory of feedback control, which in practice corresponds to evaluating new solutions (obtained from a parameter found through trial-and-error) by the modelling program. The methodology was applied to a rectangular channel geometry, with the aim of defining the curved sides of the adapter by minimizing the effects of extrudate swell.

Finally, some authors considered the use of multiple objectives during the optimization. Nóbrega et al. [147,148,149] and Carneiro et al. [150] proposed an optimization methodology based on the simplex method to design a rectilinear profile die. In the example studied, the flow channel comprises a parallel zone, a pre-parallel zone, a transition zone, and an adapter, but only the first two were considered in the design. Two decision variables were used, the length and thickness of the parallel zone, and the objective function corresponds to the weighted sum of the flow balance and of the length/thickness ratio of the zones to be optimized. The authors compared the performance of the non-linear Simplex method with a trial-and-error procedure, but no conclusion about the best method was clearly reported [151]. The optimization results were assessed experimentally [152,153]. Zhang et al. [154] applied a gradient method based on a sensitivity analysis of a response surface obtained from 3D numerical modelling relating the objective function with the design variables, to optimize an L-shape profile die. The design variables were the parameters of spline curves based on eight control points, and the objective function was the weighted sum of maximization of flow balance and extrudate-swell homogeneity and minimization of points displacement and dimensional tolerance.

**Table 5:** Previous publications on the optimization of profile dies (KP—key points (see text); MP—mesh parameterization; GP—geometry parameterization).

Objective Function	Optimization Algorithm	Modelling Approach	Decision Variables	Other Characteristics	Authors (Year) Reference
SO	Empirical	3D-N	GP	IEP	Legat and Marchal (1993) [127]
SO	Empirical	3D-N	GP	IEP	Tran-Cong and Phan-Thien (1988) [129]
SO	Empirical	A	GP		Hurez et al. (1996) [130]
SO	Empirical	3D-N	GP		Švábík et al. (1999) [131]
SO	Empirical	3D-N	GP	IEP	Gifford (2003) [132]
SO	Empirical	3D-N	GP(3)		Rezaei Shahreza et al. (2010) [133]
SO	Simplex	3D-N	GP		Coupez et al. (1999) [134]
SO	Regression	3D-N	MP		Ready and Schaub (1999) [135]
SO	Regression	3D-N	GP(22)		Elgeti et al. (2012) [136]
SO	Regression	3D-N	GP(171)	IEP	Pauli et al. (2013) [137]
SO	Gradient	3D-N	MP		Sienz et al. (1998, 2010) [138,139]
SO	Gradient	3D-N	GP		Szarvasy et al. (2000) [140]
SO	ES	3D-N	MP		Sienz et al. (1999) [141]
SO	Gradient	2D-N	KP		Ettinger et al. (2004, 2004) [142,143]
SO	Gradient	2D-N	KP(2-46]		Sienz et al. (2012) [144]
SO	SA	3D-N	GP(3)		Yilmaz et al. (2014) [145]
SO	Feedback Control	3D-N	GP	IEP	Spanjaards et al. (2021) [146]
WS(2)	Simplex	3D-N	GP		Nóbrega et al. (2002, 2003) [147,148,149]
WS(2)	Simplex	3D-N	GP		Carneiro et al. (2004) [150]
WS(4)	Gradient	3D-N	GP(8)		Zhang et al. (2019) [154]

#### 4.4.4. Calibrators

The design of calibrators is linked to that of profile dies, as they must assure that the cross-section of the extrudate stands within the defined tolerances. For that purpose, the calibrator should cool the extrudate contour uniformly until an outer layer of polymer has solidified and so its geometry is preserved. Thus, the design of calibrators involves the definition of the number and length of the units to be used, and for each, the number, location, and diameter(s) of the cooling channels. Table 6 identifies the previous optimization studies on this topic.

Fradette et al. [155] seems to have pioneered the scientific design of calibrators for profile extrusion. The strategy included a modelling routine (3D numerical modelling), an objective function (the weighted sum of minimizing the cooling time and maximizing the cooling uniformity), decision variables (48 variables defining locations and diameter of 16 cooling channels), and optimization algorithm (gradient optimization).

Nóbrega and Carneiro [156] used the simplex method to optimize a calibration system comprising three units separated by two annealing zones, with the results being obtained by a 3D numerical modelling code. The system was defined by eight geometry-related decision variables (length of calibrators and of the annealing zones, temperature of the cooling fluid in each calibrator). Subsequently [157], the number of calibrators was taken as additional decision variable and a MOEA with two objectives (minimization of the final extrudate average temperature and of the corresponding standard deviation). Duan and Zhang [158] optimized the location and diameter of the cooling channels, considering the weighted sum of two objectives (maximizing the cooling uniformity and of efficiency), but little detail was given on the optimization procedure. Finally, Ren et al. [159] applied EA to optimize a calibrator based on 3D numerical simulations, assuming the weighted sum of two objectives (maximization cooling uniformity and efficiency), with the aim to define the location and diameter of a variable number of cooling channels.

**Table 6:** Previous publications on the optimization of calibrators for extruded profiles.

Objective function	Optimization Algorithm	Modelling Approach	Decision Variables	Other Characteristics	Authors (Year) Reference
SO	Simplex	3D-N	GP(5)	-	Nóbrega and Carneiro (2005) [156]
AS(2)	Empirical	3D-N	GP(n)	-	Duan and Zhang (2014) [158]
AS(2)	Gradient	3D-N	GP(48)	-	Fradette et al. (1996) [155]
AS(2)	EA	3D-N	GP(n)	-	Ren et al. (2010) [159]
MO	EA	3D-N	GP(8)	-	Nóbrega et al. (2008) [157]

## 5. Conclusions

This review discussed the application of optimization methods to solve real problems in extrusion, namely for single- and twin-screw extruders, extrusion dies, and calibrators. It was shown that equating processing challenges as optimization problems is much more efficient than relying on empirical knowledge, or in the use of simulation tools on a trial-and-error basis.

Regardless of the specific processing routine being analysed, it is evident that there is a strong interdependence between the objective function (i.e., the system performance), the optimization algorithm, and data collecting (i.e., experimental or computational data). Selecting a specific optimization algorithm depends on the features of the problem and whether the goal is to optimize one or several objectives. Aspects such as the scarcity of data, the possibility of generating data during the optimization, as well as the time required to obtain such data must be taken into consideration as well.

The second part of this review will focus on the application of optimization approaches to moulding processes (injection and blow moulding, thermoforming). Trends in process optimization will be also discussed.

## Figures and Tables

**Table 3:** Previous publications on the optimization of manifold dies (manifold type: CH-Coat Hanger, TCH-Tapered Coat Hanger, Blow–blow moulding).

Objective Function	Optimization Algorithm	Modelling Approach	Decision Variables	Other Characteristics	Authors (Year) Reference
Not defined	Empirical	1D-A	DG	Various dies	Rakos and Sebastian (1990) [85]
SO	Empirical	1D-A	DG(1)	CH	Matsubara (1979, 1980) [86,87]
SO	Empirical	1D-A	DG(1)	T-die	Matsubara (1980, 1988) [88,89]
SO	Empirical	1D-A	DG(3)	CH	Winter and Fritz (1986) [90]
SO	Empirical	3D-N	DG(3)	CH	Liu et al. (1988, 1994) [91]
SO	Empirical	3D-N	DG(4)	TCH, 2 cavities	Lee and Liu (1989) [92]
SO	Empirical	3D-N	DG(3)	CH	Liu et al. (1988, 1994) [93]
SO	Empirical	3D-N	DG(4)	TCH	Yu and Liu (1998) [94]
SO	Empirical	3D-N	DG(3)	CH	Na and Kim (1995) [95]
SO	Empirical	2D-N	DG(2)	CH	Huang et al. (2004) [96]
SO	Regression	1D-A	OC(1) + DG(3)	CH	Chen et al. (1997) [97]
SO	Regression	3D-N	DG(5)	CH	Razeghiyadaki et al. (2020, 2021) [98,99]
SO	SQP + Regression	3D-N	DG(1)	CH	Lebaal et al. (2006) [100]
SO	SQP + Regression	3D-N	DG(4)	CH	Lebaal et al. (2009) [101]
SO	SQP + Regression	3D-N	OC(3) + DG(1)	CH	Lebaal et al. (2010) [102]
SO	SQP + Regression	3D-N	DG(4)	CH (wire)	Lebaal et al. (2012) [103]
SO	Gradient	3D-N	DG(2)	CH	Smith et al. (1998, 1998) [104,105]
SO	Gradient	3D-N	OC(1) + DG(2)	CH	Smith (2003) [106]
SO	Gradient	3D-N	DG(811)	CH, Robustness	Smith (2003) [107]
SO	Gradient	3D-N	DG(9)	CH	Sun and Gupta (2004) [108]
SO	Gradient	3D-N	DG(5)	CH, Restrictor	Bates et al. (2003) [109]
SO	Regression + Gradient + EA	3D-N	DG(5)	CH, Restrictor	Siens et al. (2006) [110]
SO	EA	3D-N	DG(n)	CH	Michaeli and Kaul (2004) [111]
SO	EA	3D-N	DG(2)	CH	Meng and Zhao (2011) [112]
SO	EA	3D-N	DG(4)	Slot die	Sun and Wang (2010) [113]
SO	EA	3D-N	DG(2)	Blow: 2-CH	Meng et al. (2009, 2012) [114,115]
AS(2)	Regression	3D-N	DG(3)	CH	Han and Wang (2012) [116]
AS(n)	Gradient	3D-N	OC(1) + DG(2)	CH, Robustness	Smith and Wang (2004) [117]
AS(n)	Gradient	3D-N	OC(1) + DG(2)	CH	Smith and Wang (2005) [118]
AS(n)	SQP	3D-N	OC(1) + DG(2)	CH	Wang and Smith (2006) [119,120]
AS(3)	EA	3D-N	OC() + DG()	CH	Zhang et al. (2020) [121]
MO(2)	DOE, RSM, EA	3D-N	DG(3/8/12)	CH	Lee et al. (2015) [122]
MO(2)	EA	3D-N	DG(3)	CH	Han and Wang (2012) [123]
AS(2) & MO(2)	Regression + EA	3D-N	DG(1)	Blow: 2-CH	Han and Wang (2014) [124]

**Table 4:** Previous publications on the optimization of mandrel.

Objective Function	Optimization Algorithm	Modelling Approach	Decision Variables	Other Characteristics	Authors (Year) Reference
SO	Regression	2D-N	DG(4)	-	Huang (1998) [125]
MO(2)	EA	3D-N + ANN	DG(3)	-	Mu et al. (2010) [126]
